# Case Report: Recurrent Placental Abruption During Pregnancy in a Patient With Pseudoexstrophy

**DOI:** 10.3389/fmed.2021.619322

**Published:** 2021-01-26

**Authors:** Benshuo Cai, Yuheng Guan

**Affiliations:** ^1^Department of Obstetrics and Gynecology, Shengjing Hospital of China Medical University, Shenyang, China; ^2^Department of Radiology, The First Hospital of China Medical University, Shenyang, China

**Keywords:** pregnancy, cesarean section, case report, placental abruption, pseudoexstrophy

## Abstract

**Background:** Pseudoexstrophy is a rare variant of the exstrophy-epispadias complex, which comprises musculoskeletal defects associated with bladder exstrophy without any urinary tract defects. However, only a few pregnancy complications have been reported in patients with pseudoexstrophy.

**Case Presentation:** This report presents the case of a woman with pseudoexstrophy, who survived recurrent placental abruption during the second trimester of her pregnancy. The patient presented with a bicornuate uterus and survived placental abruption twice, which may have resulted from the malformation of the uterus. Placental abruption occurred at 20 weeks during her first pregnancy, and because she was already in labor, uterine contraction was augmented until vaginal delivery was achieved. The second pregnancy, however, could not be terminated quickly enough; therefore, a cesarean section was performed to save the patient's life.

**Conclusions:** Our study makes a significant contribution to the literature although pregnancy complications have been reported in patients with pseudoexstrophy. Our findings show that in female patients with pseudoexstrophy who are or wish to become pregnant, detailed imaging studies must be performed to identify any deformities of the pelvis or reproductive organs, in order to make a pregnancy-related risk assessment. Our experience also indicates that if surgery is inevitable, the obstetrician must be more careful when entering the abdominal cavity during the surgery to avoid secondary injury. Furthermore, the peritoneum and fascia layers must be sutured more firmly when closing the abdomen to avoid an abdominal wall hernia, because of the lack of abdominal muscle and fat tissue in such patients.

## Introduction

Exstrophy of the bladder is a rare congenital birth defect that occurs in roughly one in 30,000–50,000 livebirths. Other variants of exstrophy are much rarer, accounting for 8% of the total cases (1). Pseudoexstrophy is one such rare variant of the exstrophy-epispadias complex, which was first described in 1954 by Hejtmancik et al. (2). It comprises of musculoskeletal defects associated with bladder exstrophy without any urinary tract defects. Patients with pseudoexstrophy usually present with a low-set or pubic umbilicus (3, 4), although many are symptom-free for life. Only a few pregnancy complications, however, have been reported in patients with pseudoexstrophy. Herein, we present the case of a woman with pseudoexstrophy, who survived recurrent placental abruption during the second trimester of her pregnancy.

## Case Description

A 26-year-old woman, gravida 2 para 0, was admitted to the hospital at 25 weeks of gestation because of “sudden heavy vaginal bleeding.” Her first pregnancy had been terminated by medically induced labor following fetal demise resulting from placental abruption. On admission, the patient presented with shock, with a blood pressure of 70/47 mmHg, and a heart rate of 105 bpm. Physical examination showed a low-set umbilicus and scarring around the umbilicus. Local skin on the lower abdomen presented with flaky scarring, under which the muscle and fat tissue were absent, the pubic bones were widely separated, the clitoris was separated, and the anterior joint of the labia demonstrated epispadias. Regarding these anomalies, the patient reported that she had never visited a doctor before, and her medical history was unknown. Internal examination via speculum demonstrated a hypertrophic cervix about 2 cm from the vaginal orifice, which was neither effaced nor open, with persistent bleeding. Three-dimensional abdominal ultrasound showed a single intrauterine pregnancy with placental thickening; additionally, a 8.2 × 3.8 × 1.8 cm mass was seen under the placenta, the maternal rectus abdominis was separated, and the thickness of the upper abdominal wall was about 0.9 cm, whereas the thickness of the lower abdominal wall was 0.3 cm, under which the muscle and fat layers were absent.

**Figure 1:**
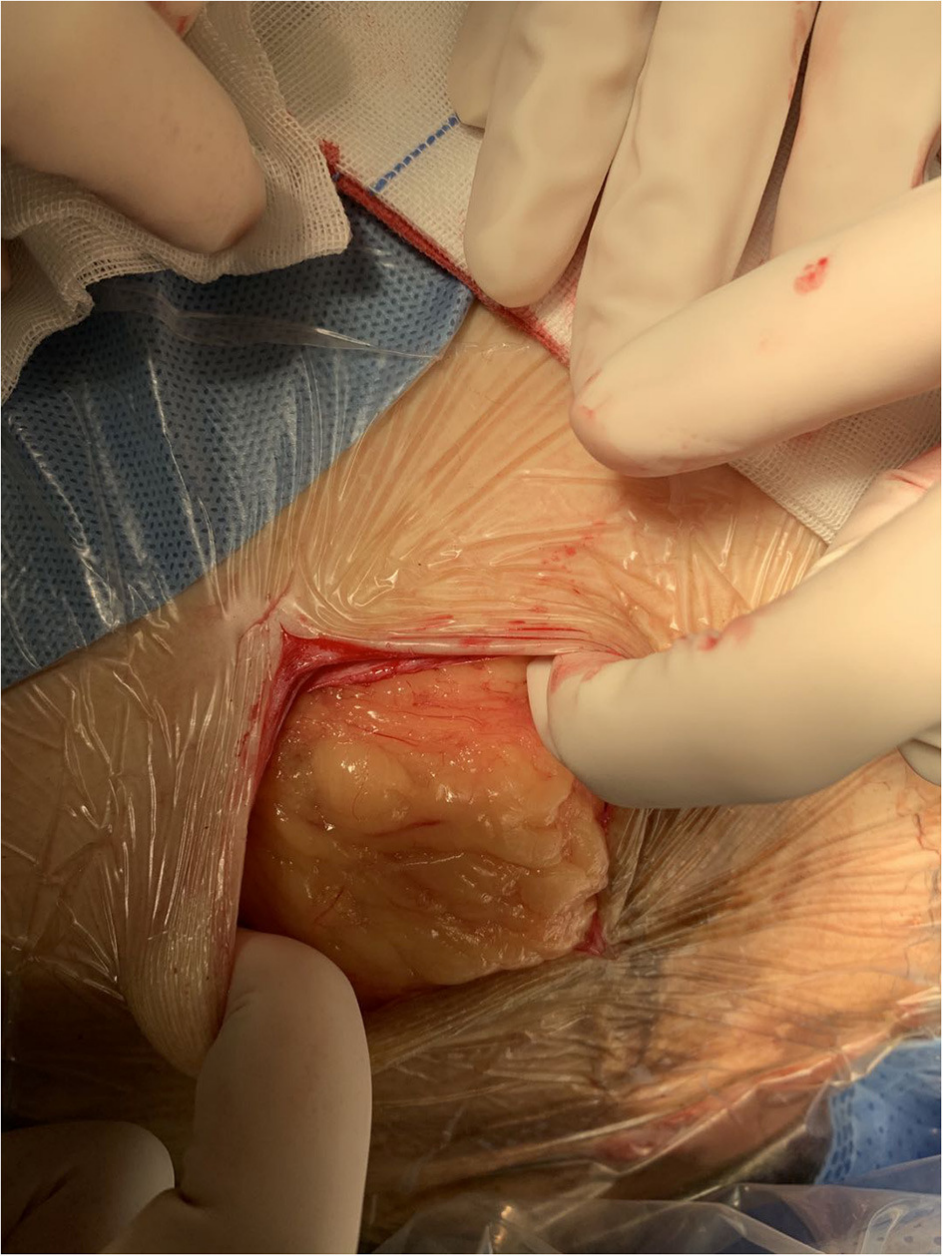
Image showing the absence of subcutaneous fat and muscle tissue.

**Figure 2:**
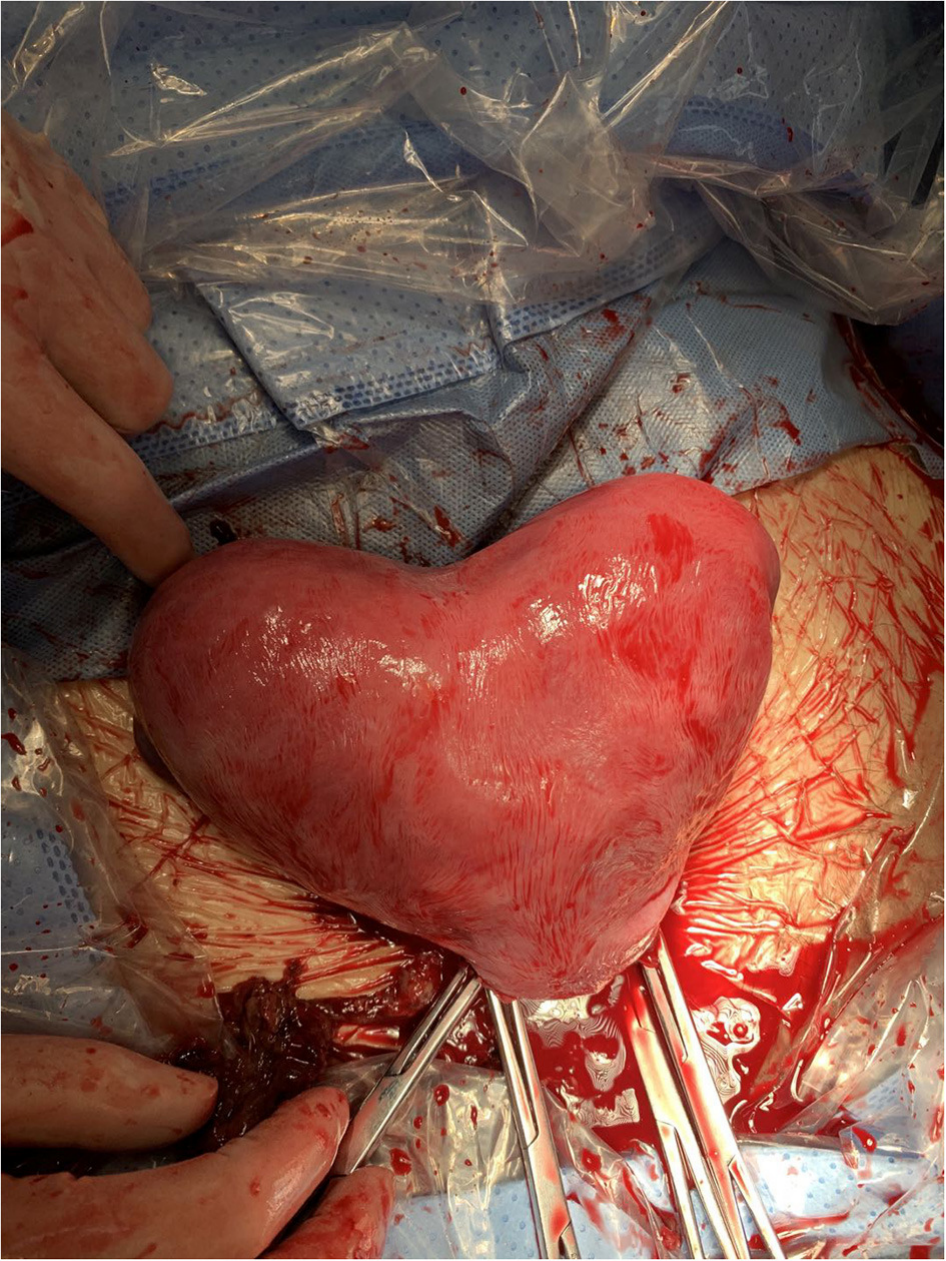
Image showing the bicornuate uterus.

**Figure 3:**
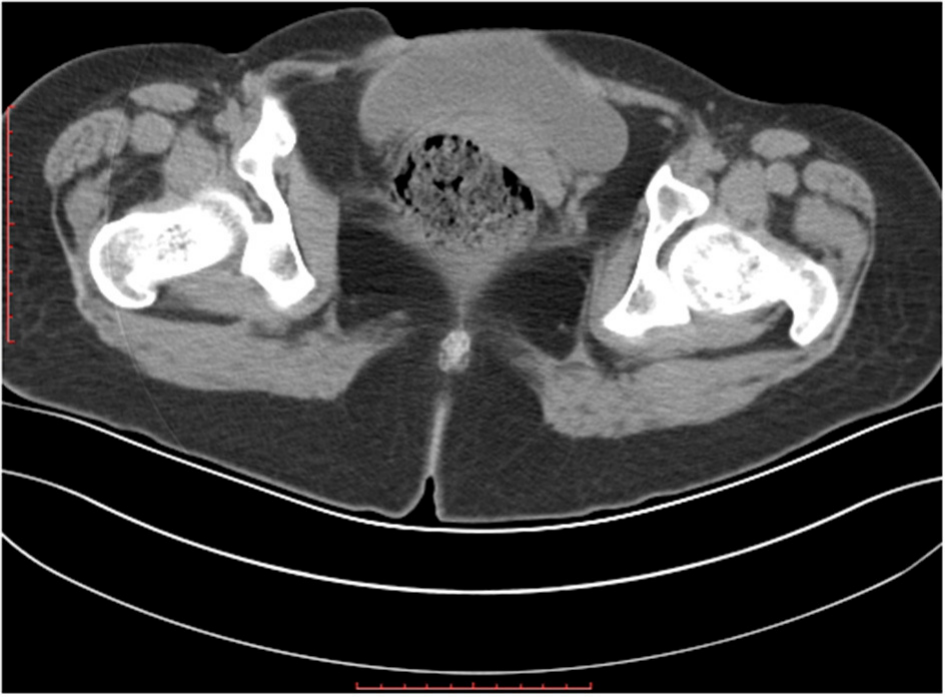
Computed tomography showing the large fascial defect on the axial slices.

**Figure 4:**
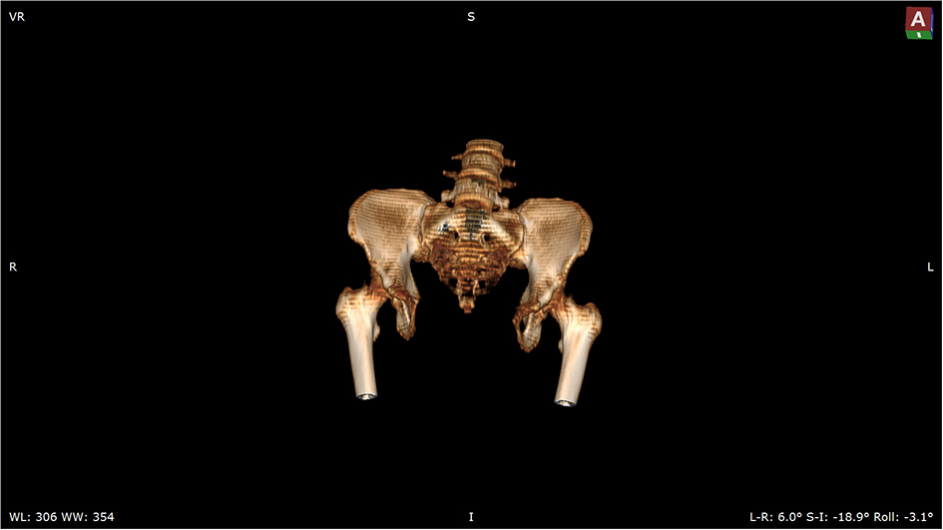
Three dimensional-computed tomography reveals the separated pubic symphysis.

**Figure 5:**
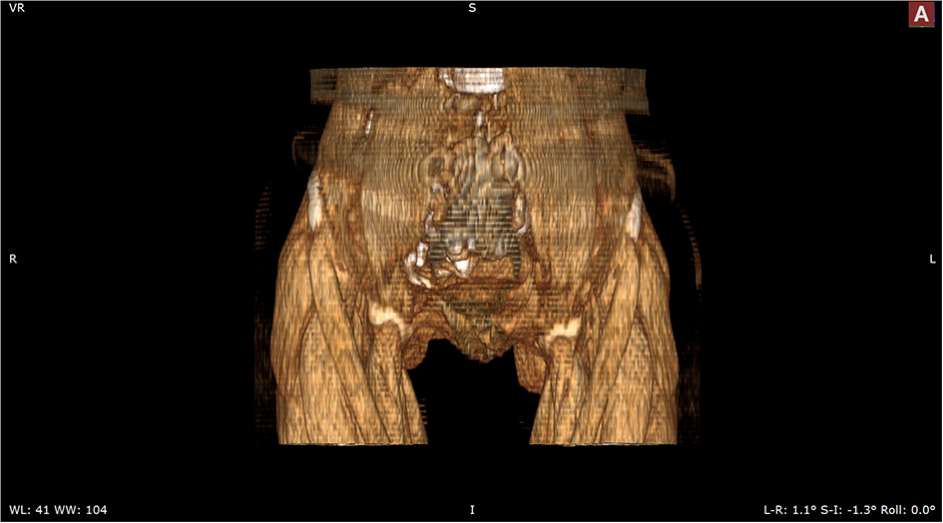
Three dimensional-computed tomography reveals the separated rectus muscles.

The patient was admitted with a diagnosis of placental abruption, hemorrhagic shock, pseudoexstrophy, epispadias, and diastasis of the symphysis pubis. After obtaining an informed consent from the patient, a cesarean section was performed. A 6 cm long longitudinal incision was carefully made in the skin at a three-horizontal-fingers distance above the upper edge of the mons pubis. As mentioned earlier, the subcutaneous fat and muscle tissue were absent, the fascia layer and peritoneum layer fit closely, and the thickness of the whole lower abdominal wall was 0.3 cm (Figure 1). The bladder was close to the lower abdominal wall and appeared intact. A demised female infant was delivered through a low transverse incision in the uterus. When checking the placenta, a partial placental abruption was evident, with a separation area of 5 × 5 cm. The uterus appeared bicornuate and both adnexa appeared normal (Figure 2). After suturing the uterine incision regularly, a continuous suturing method with an absorbable thread was performed to suture both the fascia and peritoneum together. The patient was discharged on her fourth post-delivery day. Pelvic computerized tomography (CT) performed 42 days after surgery showed that the muscle layer of the abdominal wall was absent, the space between bladder and abdominal wall was not clearly demarcated (Figure 3), and diastasis of the symphysis pubis and the rectus muscles were also visible (Figures 4, 5).

## Discussion

The exstrophy-epispadias complex represents a spectrum of birth defects, which include epispadias, pubic diastasis, bladder exstrophy, and cloacal exstrophy. The etiology of the exstrophy-epispadias complex is still unclear, but is believed to result from persistence or overdevelopment of an abnormal infraumbilical cloacal membrane, preventing medial migration, and ingrowth of the mesenchymal tissues (1). The exstrophy-epispadias complex is often associated with anomalies of the genitalia due to the fact that embryogenesis of the urinary tract and genitalia are closely connected. Blakeley (5) and Stanton (6) reported that of 79 patients, around 50% with bladder exstrophy presented with an anomaly of the genitalia that could affect their pregnancies. Some researchers believe that bladder exstrophy increases the risk of miscarriage during pregnancy (7–14).

In our case, the patient presented with a bicornuate uterus and survived placental abruption twice, which may have resulted from the malformation of the uterus. Placental abruption occurred at 20 weeks during her first pregnancy, and because she was already in labor, uterine contraction was augmented until vaginal delivery was achieved. The second time, under our care, however, the patient was in shock due to heavy vaginal bleeding. As the pregnancy could not be terminated quickly enough, a cesarean section was performed to save the patient's life. Assuming that the bladder may be close to the abdominal wall, a high located midline longitudinal incision was performed very gently, in order to avoid damaging the bladder when entering the abdomen, intestines, or other organs that could potentially be damaged.

Pseudoexstrophy is a rare congenital anomaly that is often accompanied by multiple genital malformations (15), which result in many obstetric complications in female patients, including spontaneous abortion, prematurity, malpresentations, and genital prolapse (5–10, 16–21). There is still controversy about the optimal method of delivery for pregnant patients with pseudoexstrophy. Some observations prove that a vaginal delivery is relatively easy due to the pelvic anatomic condition, although the probability of prolapse increases. However, several studies revealed that a cesarean section did not always reduce the risk of a prolapse (16, 17). Thus, obstetric conditions should be taken into consideration when making the decision regarding the delivery method.

In conclusion, in female patients with pseudoexstrophy who are or wish to become pregnant, a multi-disciplinary team (including obstetricians, radiologists, urologists, general surgeons, and pediatricians) management approach is necessary from diagnosis to pre-conception up to prenatal, peri- and post- partum (10, 11). Detailed imaging studies must be performed to identify deformities of the pelvis or reproductive organs, if any, in order to make a pregnancy-related risk assessment, and any possible adverse prognosis should be discussed before pregnancy, when possible. Otherwise, routine obstetric examinations should be performed during the pregnancy and the delivery method must be decided before the labor begins. If surgery is inevitable, the obstetrician should be extra careful when entering the abdominal cavity. During the surgery, a small incision should be performed at least 1–2 cm above the area where muscle and fat layers are absent, then the incision should be gradually and gently extended downward, to avoid secondary injury. Furthermore, the peritoneum and fascia layers should be sutured more firmly while closing the abdomen to avoid an abdominal wall hernia, because of the lack of abdominal muscle and fat tissue in such patients. If the tension of the abdominal wall is too high, a patch could be used. The urologist and general surgeon should be informed during the surgery, if necessary (10, 11). In addition to routine obstetric follow-up at 42 days after delivery, she was also recalled for abdominal incision follow-up to check on wound healing and rule out incisional hernia. Ultrasonography and CT were also necessary. Preconceptional consultation is recommended for such a patient before she embarks on her next pregnancy.

## Data Availability Statement

The original contributions presented in the study are included in the article/supplementary material, further inquiries can be directed to the corresponding author/s.

## Ethics Statement

Written informed consent was obtained from the individual(s) for the publication of any potentially identifiable images or data included in this article.

## Author Contributions

BC collected the patient's information, performed the surgery, and wrote the manuscript. YG analyzed the computed tomography images. All authors approved the final manuscript.

## Conflict of Interest

The authors declare that the research was conducted in the absence of any commercial or financial relationships that could be construed as a potential conflict of interest.

## References

[1] Marshall VF Muecke EC Variations in exstrophy of the bladder. J Urol. (1962) 88:766–96. 10.1016/S0022-5347(17)64883-3

[2] Hejtmancik JH King WB Magid MA. Pseudo-exstrophy of bladder. J Urol. (1954) 72:829–32. 10.1016/S0022-5347(17)67677-8 13212885

[3] Kittredge WE Bradburn C. Incomplete exstrophy of the bladder; case report. J Urol. (1954) 72:38–40. 10.1016/S0022-5347(17)67537-2 13163992

[4] Turner WR Jr Ransley PG Bloom DA Williams DI. Variants of the exstrophic complex. Urol Clin North Am. (1980) 7:493–501. 7404880

[5] Stanton SL. Gynecologic complications of epispadias and bladder exstrophy. Am J Obstet Gynecol. (1974) 119:749–54. 10.1016/0002-9378(74)90086-6 4858236

[6] Blakeley CR Mills WG. The obstetric and gynaecological complications of bladder exstrophy and epispadias. Br J Obstet Gynaecol. (1981) 88:167–73. 10.1111/j.1471-0528.1981.tb00963.x 7459306

[7] Burbige KA Hensle TW Chambers WJ Leb R Jeter KF. Pregnancy and sexual function in women with bladder exstrophy. Urology. (1986) 28:12–4. 10.1016/0090-4295(86)90172-X 3727222

[8] Clemetson CA. Ectopia vesicae and split pelvis; an account of pregnancy in a woman with treated ectopia vesicae and split pelvis, including a review of the literature. J Obstet Gynaecol Br Emp. (1958) 65:973–81. 10.1111/j.1471-0528.1958.tb08591.x 13621295

[9] Lepercq J Landowski P Tournaire M Paniel BJ. [Bladder exstrophy and pregnancy]. J Gynecol Obstet Biol Reprod (Paris). (1994) 23:628–31. 7983333

[10] Deans R Banks F Liao LM Wood D Woodhouse C Creighton SM. Reproductive outcomes in women with classic bladder exstrophy: an observational cross-sectional study. Am J Obstet Gynecol. (2012) 206:496 e1–6. 10.1016/j.ajog.2012.03.016 22537419

[11] Dy GW Willihnganz-Lawson KH Shnorhavorian M Delaney SS Amies Oelschlager AM Merguerian PA Successful pregnancy in patients with exstrophy-epispadias complex: A University of Washington experience. J Pediatr Urol. (2015) 11:213 e1–6. 10.1016/j.jpurol.2015.04.019 26092092

[12] Giron AM Passerotti CC Nguyen H Cruz JA Srougi M. Bladder exstrophy: reconstructed female patients achieving normal pregnancy and delivering normal babies. Int Braz J Urol. (2011) 37:605–10. 10.1590/S1677-55382011000500006 22099272

[13] Mathews RI Gan M Gearhart JP. Urogynaecological and obstetric issues in women with the exstrophy-epispadias complex. BJU Int. (2003) 91:845–9. 10.1046/j.1464-410X.2003.04244.x 12780845

[14] Borzi P Kimble R He Y successful pregnancy outcome and surgical approach in women with re-paired bladder exstrophy or cloacal exstrophy–experience from a quaternary paediatric and adolescent gynaecology centre in Australia. J Pedia Cong Disord. (2016) 1:1–5.

[15] Muecke EC. The role of the cloacal membrane in exstrophy: the first successful experimental study. J Urol. (1964). 92:659–67. 10.1016/S0022-5347(17)64028-X 14241195

[16] Krisiloff M Puchner PJ Tretter W Macfarlane MT Lattimer JK. Pregnancy in women with bladder exstrophy. J Urol. (1978) 119:478–9. 10.1016/S0022-5347(17)57522-9 650748

[17] Ikeme AC. Pregnancy in women after repair of bladder exstrophy. two case reports. Br J Obstet Gynaecol. (1981). 88:327–8. 10.1111/j.1471-0528.1981.tb00990.x 7470425

[18] Peneau M Body G Lansac J Berger C Lanson Y Zephir D. [Bladder exstrophy and pregnancy. Apropos of 2 cases]. Ann Urol. (1985) 19:47–52. 3985573

[19] Kennedy WA II Hensle TW Reiley EA Fox HE Haus T. Pregnancy after orthotopic continent urinary diversion. Surg Gynecol Obstet. (1993) 177:405–9. 8211586

[20] Barrett RJ II Peters WA III. Pregnancy following urinary diversion. Obstet Gynecol. (1983) 62:582–6. 6621946

[21] Dewhurst J Toplis PJ Shepherd JH. Ivalon sponge hysterosacropexy for genital prolapse in patients with bladder extrophy. Br J Obstet Gynaecol. (1980) 87:67–9. 10.1111/j.1471-0528.1980.tb04428.x 7362792

